# Herd-Level Risk Factors Associated with *Mycoplasma bovis* Serostatus in Youngstock on Irish Dairy Farms

**DOI:** 10.3390/ani14213057

**Published:** 2024-10-23

**Authors:** Marie-Claire McCarthy, Luke O’Grady, Conor G. McAloon, John F. Mee

**Affiliations:** 1 Cork Regional Veterinary Laboratory, Department of Agriculture, Food and the Marine, T12 XD51 Cork, Ireland; 2 School of Veterinary Medicine, University College Dublin, Belfield, D04W6F6 Dublin 4, Ireland; luke.ogrady@ucd.ie (L.O.); conor.mcaloon@ucd.ie (C.G.M.); 3 Teagasc, Animal and Bioscience Research Department, Dairy Production Research Centre, Moorepark, Fermoy, P61P302 Co Cork, Ireland; john.mee@teagasc.ie

**Keywords:** *Mycoplasma bovis*, cattle health, dairy heifers, herd-level risk factors, dairy herds

## Abstract

*Mycoplasma bovis* is a globally significant pathogen of cattle associated with a wide range of clinical syndromes, including respiratory disease, mastitis, arthritis, otitis, and reproductive failure. Since its detection in Ireland in 1994, *M. bovis* has become a significant contributor to morbidity and mortality in Irish cattle. This study aimed to investigate herd-level risk factors associated with *M. bovis* seropositivity in replacement dairy heifers, using data from 105 Irish dairy herds. Ten heifers per herd were sampled on three occasions: spring 2018, spring 2019, and autumn 2019. Seropositivity was evaluated using two thresholds: ≥1 positive heifer (Model ≥ 1POS) and ≥3 positive heifers (Model ≥ 3POS). *M. bovis* seropositivity varied over time, with at least one positive heifer in 50.4% (95% confidence interval (CI): 40.5–60.3) of herds in spring 2018, 35.2% (95% CI: 26.2–45.1) in spring 2019, and 45.7% (95% CI: 36.0–55.7) in autumn 2019. Herds with three or more positive heifers increased from 31.4% (95%CI: 22.7–41.2) in spring 2018 to 42.9% (95% CI: 33.2–52.9) by autumn 2019. Risk factors for *M. bovis* seropositivity included the purchase of cattle, which significantly raised the odds of seropositivity across multiple visit periods (Model ≥ 1POS: Odds ratio (OR) 3.84, *p* = 0.02; Model ≥ 3POS: OR 3.69, *p* = 0.02). Managing more than three land parcels, housing heifer calves separately from bull calves, and sharing airspace between calves and older animals also increased seropositivity risks. Conversely, more colostrum feeds reduced the risk of seropositivity (Model ≥ 1POS: OR 0.81, *p* = 0.05), while colostrum quality assessment and feeding waste milk showed a trend toward increased risk. These findings suggest the importance of robust biosecurity measures, including limiting cattle purchases, improving calf management, and enhancing colostrum feeding practices, to control the spread of *M. bovis*. This study provides valuable insights into the epidemiology of *M. bovis* in Irish dairy herds, emphasising the need for targeted biosecurity and surveillance to safeguard herd productivity.

## Simple Summary

*Mycoplasma bovis* is a significant pathogen in cattle, causing diseases such as respiratory illness, mastitis, arthritis, and reproductive failure. First detected in Ireland in 1994, it has since become a major health issue in Irish cattle herds. This study investigated the risk factors associated with *M. bovis* seropositivity in replacement dairy heifers across 105 Irish dairy herds. Ten heifers per herd were sampled during three periods: spring 2018, spring 2019, and autumn 2019. Results showed that seropositivity varied over time, with 50.4% of herds having at least one positive heifer in spring 2018, 35.2% in spring 2019, and 45.7% in autumn 2019. Risk factors for seropositivity included the purchase of cattle, managing multiple land parcels, and housing heifer calves separately from bull calves. Shared airspace between calves and older animals also increased the risk of *M. bovis* seropositivity. Conversely, feeding more colostrum reduced the odds of seropositivity. These findings highlight the importance of implementing strong biosecurity measures, improving calf management, and enhancing colostrum feeding practices to control the spread of *M. bovis* in Irish dairy herds.

## 1. Introduction

*Mycoplasma bovis* is a significant pathogen affecting cattle populations globally. Clinically, mycoplasmosis manifests in various forms, with arthritis and mastitis being prevalent in adult cattle, while pneumonia, arthritis, and otitis are primarily associated with infections in calves [1,2,3]. These infections contribute to significant economic losses in dairy and beef industries due to reduced milk production, treatment costs, and culling of infected animals [4]. For example, in the U.S., *M. bovis* respiratory infections in beef cattle, which lead to reduced weight gain and lower carcass value, are estimated to cost USD 32 million annually. Additionally, the economic losses from bovine mastitis caused by *M. bovis* may exceed those from respiratory infections, with estimates reaching up to USD 108 million per year [5]. Since its first detection in Ireland in 1994 [6], *M. bovis* has become endemic and is now considered a major cause of youngstock morbidity and mortality. For example, it has been identified as the aetiological agent in 14.3% of neonatal (0–1 month) and 13.7% of older calf (1–5 months of age) deaths attributed to respiratory disease in Ireland in 2022, respectively [7]. The herd-level prevalence of *M. bovis* in Irish dairy herds has recently been estimated at 45% using bulk tank milk (BTM) ELISA testing [8]. In that study herd seropositivity was associated with herd size, the number of contiguous farms, and geographical location. While that study provides valuable insights into *M. bovis* seroprevalence at the herd-level, research focusing specifically on Irish dairy herds—particularly regarding youngstock and replacement dairy heifers—remains limited. Additionally, using bulk tank milk as a matrix for seroprevalence studies presents several inherent limitations. While BTM ELISA testing is both cost-effective and convenient—requiring only a single pooled milk sample for large-scale herd-level screening—it has significant limitations. Most notably, it excludes non-lactating animals such as calves, heifers, sick cows, and dry cows, which may serve as substantial reservoirs of *M. bovis* infection, potentially leading to inaccurate herd-level prevalence estimates. Moreover, pooling of milk samples can dilute antibody concentrations, particularly when there are small numbers of infected cows, which reduces the test sensitivity. External factors, such as herd size, may also affect test accuracy and introduce biases in prevalence estimates. In contrast, seroprevalence studies in youngstock cohorts offer a more detailed assessment of *M. bovis* infection dynamics within a herd. These studies not only identify seroprevalence trends but also provide critical insights into the natural progression of *M. bovis* infections, such as the duration of antibody persistence following natural infection. Moreover, since adult animals may have persistent antibodies from previous infections, making it difficult to distinguish between current and past exposure, sampling youngstock avoids this issue. Their antibody response is more likely to reflect recent or ongoing infection.

In Swiss dairy herds, *M. bovis* infections were more prevalent in high-yielding herds and those with a high rate of animal movements (e.g., cattle shows or trade) [9]. In Belgian dairy herds, the absence of a dedicated calving pen and the use of a breeding bull significantly increased the risk of *M. bovis* presence in dairy herds [10]. Large herd size (greater than 100 animals), lack of quarantine procedures for newly purchased animals, and higher-than-average milk production were found to be major risk factors for *M. bovis* seropositivity in Brazilian dairy herds, managed under tropical conditions [11]. In a Japanese study, large dairy herds (greater than 200 animals) and herds that frequently purchased cattle were at higher risk of *M. bovis* infection [12]. While these studies provided seroprevalence estimates for lactating dairy cows, they did not include non-lactating animals such as replacement dairy heifers. Replacement dairy heifers constitute a key demographic in epidemiological investigations, as they can act as reservoirs for *M. bovis* and significantly influence intra-herd transmission dynamics [13]. The omission of non-lactating animals, such as heifers, from seroprevalence assessments limits the ability to thoroughly evaluate *M. bovis* transmission and its subsequent effects on herd health.

Risk factors associated with *M. bovis* infections in calves are varied and include several management practices and environmental conditions. One significant risk is the feeding of milk contaminated with *M. bovis*, such as waste milk from infected cows, which has been shown to facilitate transmission [14,15]. Additionally, the proximity of infected adult cows or calves to young calves increases the likelihood of infection. Infections frequently originate from cows with subclinical mastitis, which can shed the bacteria through milk or respiratory secretions, amplifying the risk of *M. bovis* transmission [16]. Calves housed in contaminated environments such as those with shared bedding or water sources, are also at heightened risk of exposure to *M. bovis*. Poor ventilation and overcrowding in housing facilities exacerbate this risk by contributing to the spread of respiratory infections [17]. Preweaning exposure to *M. bovis* in replacement dairy heifers significantly increases the likelihood of future pathogen shedding and disease transmission within the herd, highlighting the long-term impacts of early-life exposure [13].

Despite the reported morbidity and high herd prevalence of *M. bovis* in Ireland, the seroprevalence of this pathogen in dairy calves and youngstock remains largely unexplored. While previous studies have focused on adult cattle and lactating cows, there is a significant gap in understanding the infection dynamics among younger animals, which play a crucial role in the epidemiology of *M. bovis*. This study addresses a significant gap in the current understanding of *M. bovis* transmission by examining the seroprevalence in replacement dairy heifers during the critical rearing period (0–2 years of age). Additionally, it aims to determine herd-level risk factors associated with seropositivity in these youngstock cohorts. By focusing on this under-researched demographic, this study provides valuable insights into the spread of *M. bovis* and informs more effective control strategies for managing the infection within Irish dairy herds.

## 2. Materials and Methods

### 2.1. Herd Recruitment

In total, 120 dairy herds were recruited to a wider national longitudinal study to assess the animal health risks associated with contract-rearing in Ireland between 2018 and 2021 as described in McCarthy et al. [18]. Briefly, a national register of source dairy farms (SDF), (farmers who sent their heifers out to be contract-reared) and control farms (CF), (farmers who did not send their heifers out to be contract-reared) was generated. The 256 SDF were identified from the Irish Cattle Breeding Federation (ICBF) HerdPlus database. Control farms were matched to the SDFs by herd size, calving pattern, and geographical location. Telecontact with these farmers resulted in a database of 120 SDFs and 85 CFs. A total of 66 SDFs and 54 CFs were recruited from the database to participate in a longitudinal study of the animal health and production implications of contract-rearing [18,19,20,21,22]. The recruited farms were distributed across all 4 provinces and 19 of the 26 counties of the Republic of Ireland, with the largest density of farms located in County Cork, reflecting the distribution of the national dairy cow population. The majority of recruited herds were classified as spring-calving (92%), with the remaining herds operating a split-calving pattern (spring and autumn).

### 2.2. Farm Visit Schedule

A single cohort of heifer calves born in spring 2018 was followed until the end of their first lactation in 2021. Between spring 2018 and autumn 2019, SDFs and CFs were visited four times. The first visit occurred when heifers were approximately 1 month old, conducted on their farm of origin. Subsequent visits for home-reared heifers remained at the farm of origin, while for contract-reared heifers, visits were subsequently carried out on the contract-rearing unit. The second visit, when heifers were around 8 months old, occurred between September and December 2018. The third and fourth visits occurred when the heifers were approximately 12 months old in spring 2019 and 20 months old in autumn 2019. Approximately 6500 heifers were enrolled initially, with data available for 5532 heifers across all four visits after losses due to sales, farm drop-out, and mortality.

### 2.3. Blood Sampling

During the first (spring 2018), third (spring 2019), and fourth (autumn 2019) farm visits, blood samples were taken from 10 heifers on each farm. Heifers were sampled at random, typically with one heifer selected each time the handling facility (chute) was refilled, depending on its capacity and the size of the heifer cohort. Blood samples were not obtained at the second farm visit (autumn 2018) as the collection of other sample types, including faecal samples was prioritised at this visit.

Samples were initially obtained from young calves (<4 months old) via jugular venepuncture. At subsequent farm visits, blood samples were collected by coccygeal venepuncture from older animals. Samples were collected in plain vacutainer tubes (BD Vacutainer, BD, Langen, Germany) and stored in a cooler unit until returned to the research centre (Teagasc, Moorepark, Cork, Ireland). Samples were refrigerated for approximately 24 h post-collection, after which time serum was separated by centrifugation at 3500× *g* for 15 min at 4 °C and frozen at −20 °C pending analysis at the conclusion of all farm visits.

Serum samples were analysed by a commercially accredited laboratory (FarmLab Diagnostics, Roscommon, Ireland) using the *M. bovis* ID Screen^®^ *Mycoplasma bovis* antibody ELISA (IDVet, Montpellier, France). All analyses were performed according to the manufacturer’s instructions. The ID Screen^®^ *Mycoplasma bovis* antibody ELISA has a reported sensitivity of 95.7% and a specificity of 100%. The cut-off for a positive sample was an S/N (sample to negative control ratio) value of <0.6. Inconclusive test results were classified as negative in the data analysis. There is no *M. bovis* vaccine available in Ireland. The median age of sampled heifers was 1.5, 12.8, and 20.2 months during the first, second, and final farm visits, respectively. Details on the health status of the studied heifer population, including the detection rate of *M. bovis* in nasal swabs from calves with respiratory disease symptoms, have been previously reported by McCarthy et al. (2021) [18].

### 2.4. Biosecurity Survey

To assess the biosecurity status of study farms (and thus risk factors associated with *M. bovis*), all participating farmers were invited to complete a questionnaire relating to biosecurity and management practices on their farms in September 2018. The questionnaire was compiled as described in McCarthy et al. [20]. Briefly, the questionnaire was compiled following a systematic literature review to identify existing questionnaires with a significant biosecurity component. Approximately 30 questionnaires were assessed for suitability, but none met the study’s aims. Instead, relevant questions were compiled from web-based herd health management tools and selected published surveys. Additional questions were added following consultation with a biosecurity expert group.

The survey covered various aspects of farm management, including herd characteristics, bioexclusion practices, calving and newborn calf management, unweaned heifer management, weaned heifer management, and herd vaccination protocols (outlined in Table 1). The survey consisted of a combination of open- and closed-ended, multiple choice, and Likert scale questions. It was piloted and modified based on feedback to ensure completion within 30 to 40 min. Data processing involved inspecting responses, validating information with national databases, and coding responses for analysis. Descriptive statistics were calculated for farm characteristics and management practices.

### 2.5. Statistical Analysis

#### 2.5.1. Data Cleaning

This study employed a repeated cross-sectional design to assess herd-level seroprevalence of *M. bovis* across multiple farm visits. For each farm visit, the individual *M. bovis* ELISA test results from animals were aggregated at the herd level. Herds were classified as seropositive based on two criteria: (1) if at least one animal (out of a sample of ten) tested seropositive, and (2) if three or more animals (out of a sample of ten) tested seropositive. Therefore, the outcome variable for the analysis was binary, indicating whether the heifer cohort was seropositive or seronegative for *M. bovis*. Two models were then developed for each farm visit: Model ≥ 1POS, where a herd was considered seropositive if at least one heifer tested seropositive, and Model ≥ 3POS, where a herd was considered seropositive if at least three heifers tested seropositive.

The independent variables (n = 59) of interest were biosecurity and herd management factors gathered through the biosecurity questionnaire survey. Where possible, survey questions with multiple response options were dichotomised. All ELISA results and survey data were compiled in a Microsoft Excel spreadsheet and exported to R studio (Version 2024.04.2) for further analysis. Herds for which ELISA results were not available at all three visit periods were removed from the dataset, resulting in a final dataset of 105 herds available for analysis.

#### 2.5.2. Data Analysis

Multivariable logistic regression analysis was utilised to quantify the associations between various potential risk factors and *M. bovis* serostatus. This approach allowed for the estimation of the effect of each predictor variable while adjusting for potential confounders and covariates.

##### Multivariable Logistic Regression Models

Descriptive statistics were conducted using R studio (Version 2024.04.2). Continuous variables were examined visually for normality using histogram plots. Non-normally distributed continuous variables were log-transformed.

To identify herd-level risk factors associated with *M. bovis* seropositivity, three multivariable logistic regression models (corresponding to the three sampling periods) were built for each of the two criteria (≥1 positive animal and ≥3 positive animals); a total of six models. The outcomes of interest in the models were seropositivity in spring 1, seropositivity in spring 2, and seropositivity in autumn 2. All analyses were performed at the herd level. By concentrating on herd-level risk factors, this study aimed to provide actionable recommendations that could be implemented at the farm-level, potentially offering more effective and scalable solutions for managing *M. bovis* compared to focusing on individual animal-level factors. Potential risk factors were first screened for association with *M. bovis* serostatus at a univariable level. Variables with a *p* value of approximately 0.2 were brought forward to the multivariable analysis. Pearson correlation coefficients were used to assess the correlation between predictor variables. Pairs of highly correlated variables were identified using a correlation matrix (i.e., with a correlation coefficient of >0.8). To avoid bias and overfitting, the final variable selection was informed by biological relevance and causal relationships. Variables with strong biological or causal significance were prioritised. Missing data were removed before building the initial multivariable model resulting in a reduction in the number of herds available for the final multivariable model (see Table 7 for details of the number of herds for which a complete dataset was available for each visit period).

Multivariable forward stepwise models were then constructed for the three sampling periods of interest. Variables were added to the model based on their significance until adding more variables did not significantly improve the model fit (based on reduction of AIC). Backward stepwise selection was then conducted, resulting in the inclusion of the same variables, indicating robustness in the selection process.

Potential two-way interactions between variables were evaluated by incorporating interaction terms into the logistic regression model, and their significance was assessed using *p* values, with a threshold of ≤0.05 considered indicative of a statistically significant interaction. There were no biologically meaningful interactions evident. A significance level of 5% was used in all models.

To evaluate the performance of the models, McFadden’s R-squared was used. This metric assesses how much better the fitted model is compared to a null model (i.e., a model without predictors), which serves as a reference point for comparison. Higher McFadden’s R-squared values indicate a better model fit, suggesting that the model more effectively accounts for the variability in *M. bovis* seropositivity.

## 3. Results

### 3.1. Descriptive Statistics

In total, *M. bovis* ELISA results were available for 105 herds across all three farm visit periods. The median herd size was 141 cows (range 60–633 cows) with a median heifer cohort size of 41 heifers (range 10–137 heifers). The median age of heifers at each visit period is outlined in Table 2. The median age of the randomly selected sampled heifers was comparable to that of their herd mates within each herd at each visit, ensuring that the sampled animals were representative of the entire heifer cohort on the farm.

Whenever possible, 10 heifers were sampled per herd during each visit period. However, there was a slight variation in the number of samples collected at each visit due to fluctuations in the heifer cohort size at the time of sampling. This variation was particularly evident during the first visit period in spring 2018 when a proportion of the heifer cohort had not yet been born. Despite these fluctuations, 10 samples were successfully collected in 89%, 88%, and 92% of farms during visit periods 1, 2, and 3, respectively. The distribution of herds with at least one *M. bovis* seropositive heifer and three of more seropositive heifers is shown in Table 3.

In total, approximately 3200 samples were collected across all three farm visit periods. The distribution of sampling frequency at the individual heifer level was as follows; 69% of the heifers were sampled once (n = 2218), 13.4% were sampled twice (n = 432) and 1.7% were sampled three times (n = 54). The mean *M. bovis* apparent seroprevalence (within the positive herds) during each visit period is outlined in Table 4.

**Figure 1:**
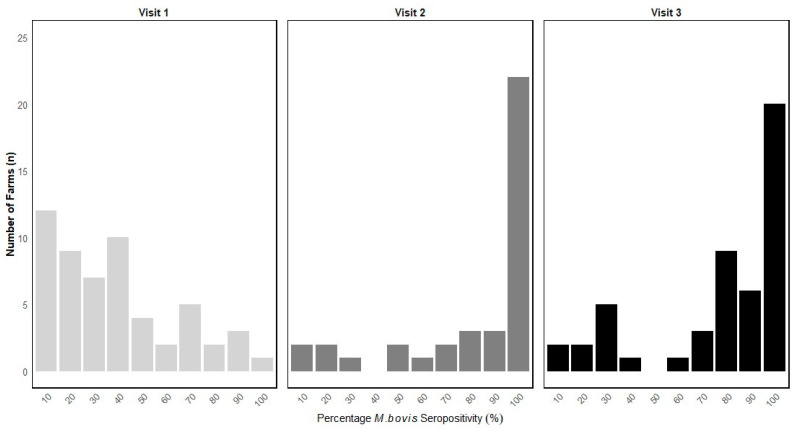
Distribution of herds (n = 105) by percentage *M. bovis* seropositivity across three farm visit periods between spring 2018 and autumn 2019.

The distribution of farms by *M. bovis* seropositivity percentage by farm visit period is demonstrated in Figure 1.

Animal-level apparent seroprevalence is demonstrated in Table 5.

The variation in the herd *M. bovis* seroprevalence across the three visit periods using the two positive threshold values of (≥1 positive heifer; ≥3 positive heifers) is outlined in Table 6.

### 3.2. Univariable Models

Univariable analysis was conducted initially to investigate the association between biosecurity and farm management practices and herd-level *M. bovis* seropositivity at each of the three visit periods (Table S1, Supplementary Materials).

In spring 1 (V1), in Model ≥ 1POS, the housing of heifer and bull calves separately had a borderline association with seropositivity (estimate: 0.78, *p* = 0.06). A greater number of colostrum feeds was associated with a slight decrease in seropositivity (estimate: −0.19, *p* = 0.05). Herd size was marginally positively associated with seropositivity, suggesting that larger herds were more likely to be seropositive (estimate: 0.003, *p* = 0.05). Additionally, the presence of non-dairy animals on the farm was marginally associated with increased seropositivity (estimate: 0.89, *p* = 0.07).

In spring 2 (V2), in Model ≥ 1POS, the practice of evaluating colostrum quality showed a non-significant trend towards higher seropositivity (estimate: 0.65, *p* = 0.12), while feeding waste milk to calves also indicated a potential increase in risk (estimate: 1.07, *p* = 0.07). The purchase of cattle during 2018 was a significant risk factor (estimate: 1.00, *p* = 0.05). Similar to Model ≥ 1POS, cattle purchase during 2018 (estimate: 0.83, *p* = 0.11) and farming multiple land parcels (estimate: 0.92, *p* = 0.04) were associated with increased risk of seropositivity in Model ≥ 3POS. Feeding waste milk to calves again showed a non-significant association with increased seropositivity (estimate: 0.92, *p* = 0.12).

During autumn 2 (V3), in Model ≥ 1POS, herds in which farmers assessed colostrum quality were more likely to be seropositive, with an estimate of 0.79 (*p* = 0.05). Similarly, the purchase of cattle in 2018 was significantly associated with seropositivity (estimate: 0.95, *p* = 0.04).

Several associations did not reach conventional levels of statistical significance (*p* < 0.05) at a univariable level, which may reflect limitations in this study’s statistical power due to the sample size. This potential lack of power could explain why some trends observed in the analysis did not achieve significance, despite suggestive estimates.

### 3.3. Multivariable Analysis

Variables with a *p* value of approximately 0.2 were selected for inclusion in the initial multivariable models for each visit period using the two herd seropositivity thresholds to determine herd *M. bovis* status. The results of the final multivariable models are presented in Table 7.

### 3.4. Model Performance

The comparison of McFadden’s R-squared values between Model ≥ 1POS and Model ≥ 3POS across the three visit periods demonstrates variations in model performance over time (Table 8). In visit 1 (spring 1), Model ≥ 1POS had an R-squared value of 0.11, while Model ≥ 3POS had a similar value of 0.10. During visit 2 (spring 2), Model ≥ 3POS showed a stronger fit, with an R-squared value of 0.31 compared to 0.13 for Model ≥ 1POS. By visit 3 (autumn 2), both models performed equally, each achieving an R-squared value of 0.16. This indicates that Model ≥ 3POS had the best explanatory power in Spring 2, while both models showed similar predictive performance in Visits 1 and 3.

## 4. Discussion

### 4.1. Overview

The key findings from this study demonstrated several critical insights into the seroprevalence of *M. bovis* in heifers across three distinct time points during the rearing period. This is the first Irish study focused specifically on youngstock *M. bovis* seroprevalence, offering a unique perspective that contrasts with previous studies, which have primarily focused on the adult (lactating) cow cohort [8]. Internationally, similar studies have highlighted *M. bovis* seroprevalence in confined systems [23], but this research expands the understanding of pathogen dynamics within pasture-based, predominantly spring-calving dairy enterprises. This study highlights the dynamic nature of *M. bovis* infection in cattle herds, with seroprevalence rising over time as heifers aged and were repeatedly exposed, suggesting that ongoing transmission within the herd plays a significant role in maintaining infection, which may complicate disease control efforts.

### 4.2. Herd- and Animal-Level M. bovis Seroprevalence over Time

In spring 2018, 50.4% of herds had at least one seropositive heifer but only 31.4% had three or more seropositive animals. These findings suggest that while *M. bovis* was present in these herds, the pathogen had not yet been extensively transmitted between heifers in the cohort. The presence of seropositive animals could be attributed either to natural infections occurring within the calf cohort or to the detection of maternal-derived antibodies (MDA) in these young animals. MDAs, transferred through colostrum, can provide temporary immunity to calves, protecting them from early infections or reducing the severity of infection, which may contribute to detectable antibody levels even in the absence of active infection. Therefore, the observed seropositivity might reflect a combination of early, limited infections and the transient presence of these maternal antibodies in the young heifers.

By spring 2019 (V2), the proportion of herds with at least one *M. bovis*-positive heifer decreased to 35.2%. While this could potentially reflect the successful implementation of control measures in some herds, it may also be attributed to statistical variation, given the small number of animals sampled from larger populations in each herd, which could influence the observed changes in seroprevalence. However, the consistent proportion of herds with three or more seropositive heifers (32.4%) suggests that once *M. bovis* was established, it continued to spread within the herd. During this period, mean within-herd seropositivity sharply increased from 38.3% to 81.3%, potentially reflecting the heightened susceptibility of older heifers as their exposure to the pathogen increased. Increased exposure due to environmental and management practices likely contributed to this rise in seroprevalence. Studies have shown that *M. bovis* can persist in herds despite control efforts, often spreading more readily within herds that have not maintained stringent biosecurity measures [24,25].

The increase in seroprevalence at the animal- level, from 20.1% in spring 2018 to 29.2% in spring 2019, aligns with the understanding that older heifers, now lacking the protective effect of maternal antibodies, became more vulnerable to *M. bovis* infections. As a result, the pathogen spread more widely within those herds that had not fully contained the infection, leading to a higher overall infection rate.

In autumn 2019 (V3), the increase in the proportion of herds with at least one seropositive heifer to 45.7% suggests a resurgence of *M. bovis* infections. This could be due to the introduction of new, susceptible animals or seasonal factors that enhanced transmission, such as changes in housing or weather conditions. The rise in herds with three or more seropositive heifers to 42.9% reflects more extensive within-herd transmission, which is often challenging to control once established. Despite a slight decrease in mean within-herd seropositivity to 77.1%, the persistently high levels indicate that *M. bovis* continued to circulate widely within the affected herds. The animal-level seroprevalence, which reached 36.1%, underscores the cumulative impact of ongoing exposure and the pathogen’s persistence over time.

This pattern is consistent with findings from other studies that highlight the difficulty in controlling *M. bovis* once it becomes endemic in a herd. Infections can persist and spread due to various factors, including the introduction of new animals, environmental stressors, and inadequate biosecurity measures. For instance, studies have shown that *M. bovis* can remain latent in some animals, re-emerging under stress or immunosuppression, which complicates eradication efforts [24,26].

Overall, the findings demonstrate that *M. bovis* seroprevalence increased both within herds and among individual animals as the heifers aged. The consistent increase in the number of heavily infected herds (with three or more positive animals) highlights the persistent challenge of controlling *M. bovis* and the need for continuous and adaptive management strategies. These results emphasise the importance of considering both age-related immunity and the dynamics of within-herd transmission when interpreting seroprevalence data and developing control measures for *M. bovis* in cattle populations. Given these trends, further investigation is warranted to better understand the underlying factors driving increased *M. bovis* transmission and to refine intervention strategies.

### 4.3. Risk Factors Associated with M. bovis Seropositivity in Replacement Heifers

This study utilised two distinct models to systematically evaluate risk factors associated with *M. bovis* seropositivity at three distinct time points during the heifer-rearing period. The results from both Model ≥ 1POS and Model ≥ 3POS provide critical insights into the epidemiology and management of *M. bovis* within dairy herds.

Number of land parcels.

An increased number of land parcels (four or more) farmed during 2018 was a significant risk factor for *M. bovis* seropositivity in both models across multiple visit periods. This association suggests that fragmented land management contributes to increased *M. bovis* transmission and infection rates within herds. These findings are consistent with those reported in an Irish study by McAloon et al. [8] who found that the number of contiguous farms was a risk factor for *M. bovis* bulk tank milk seropositivity in the lactating cow cohort. Each additional land parcel adds more shared boundaries and potential points of contact between cattle on adjoining farms, increasing the likelihood of interaction with multiple herds. This proximity can facilitate the spread of airborne and contact pathogens, especially when biosecurity measures are inconsistent across farms.

Land fragmentation is a prevalent characteristic of Irish dairy farms, with an average of six land parcels per farm, according to a study of 900 dairy farms [27]. Many of these farms are spread across multiple, non-contiguous parcels of land. In Bradfield’s study, around 59% of farms managed 6 or fewer parcels, while 41% had between 7 and 22 distinct parcels. This fragmentation complicates farm operations by increasing the frequency of movement of animals, machinery/equipment (fomites), and workers between land parcels. This increased movement can facilitate the transmission of *M. bovis* between management groups. Several studies have shown that increased contact between different animal groups and the use of shared equipment without proper biosecurity measures can lead to higher risks of *M. bovis* transmission [4,10,23]. Additionally, managing multiple parcels of land increases the complexity of biosecurity protocols. Insufficient separation between infected and uninfected animals and the challenges of maintaining appropriate hygiene standards across different locations can further exacerbate the spread of *M. bovis* [28].

Purchase of cattle.

Purchase of cattle was a consistent factor for *M. bovis* seropositivity in both models, particularly in spring 2 and autumn 2. These findings agree with those of several studies which have demonstrated that purchased cattle act as a source of new *M. bovis* infections in dairy herds [10,12,29,30]. In a risk factor study by Burnens et al. [31], the purchase of animals was the only variable significantly associated with the *M. bovis* serological status of 51 dairy herds in Switzerland. Carrier cattle without clinical signs are the primary vectors for introducing *M. bovis,* often leading to varying transmission dynamics across herds. Some herds experience immediate clinical outbreaks, while others face delayed transmission events. The risk of *M. bovis* seropositivity increases significantly when animals are sourced from multiple herds, compared to single-source or closed herds [8]. Since the removal of EU milk quotas in 2015, Ireland’s dairy sector has undergone considerable growth, with the national dairy herd expanding by approximately 45%, from 1.2 million cows in 2015 to nearly 1.6 million by 2021, accompanied by a 60% increase in milk production [32].

When herds expand through the purchase of animals, they introduce a significant biosecurity risk, as carrier cattle without clinical signs are a known vector for *M. bovis* transmission [31]. Of the herds enrolled in the current study, 93% had undergone expansion between 2013 and 2018, many by purchasing cattle, which likely increased their exposure to *M. bovis* compared to non-expanding, closed herds.

Therefore, the most effective way to prevent *M. bovis* infections is to maintain a closed herd [33]. Where this is not feasible, purchased animals should be tested and quarantined before introduction to the herd. Prior to the purchase of lactating cows, it is recommended that milk samples should be tested for *M. bovis* using culture, PCR, or ELISA. Additionally, calf health records should be reviewed for any history of *M. bovis*-related diseases, such as pneumonia or otitis media [24].

Other (non-dairy) animals are kept on the farm.

The presence of non-dairy animals (suckler beef cattle, finishing beef cattle, pigs, poultry, sheep, horses, or goats) on the farm was a significant risk factor for *M. bovis* seropositivity in replacement heifers in Model ≥ 3POS during the spring 1 visit period (V1). The odds ratio (OR 4.13) indicates that farms with non-dairy animals are substantially more likely to be *M. bovis* seropositive. While this finding has not been reported in other studies relating to *M. bovis*, a Norwegian study identified that the presence of “other animal traffic,” including common animal housing and interaction between different animal types, was associated with higher risks of BVD in dairy herds [34]. Further to this, Correa-Valencia et al. [35] demonstrated that mixed farming enterprises had increased odds of infection with *Mycobacterium avium* subsp. *paratuberculosis* (MAP). A possible explanation for the increased seropositivity on mixed farm enterprises is that specialist dairy-only enterprises may maintain a higher level of control over biosecurity due to a singular focus on one species. These farms may have better implementation of preventative measures for dairy-specific diseases such as mastitis. Furthermore, dairy-only farms are less likely to experience interspecies disease transmission, reducing the overall disease burden on these farms.

Individual farm management practices.

Feeding waste milk (unpasteurised mastitic milk and milk from antibiotic-treated cows) to calves was identified as a potential risk factor for *M. bovis* seropositivity. While the association was not statistically significant, there was a consistent trend toward higher seropositivity in both spring 2 (V2) and autumn 2, indicating a potential but inconclusive association. This practice is linked to an elevated risk of transmitting several pathogenic agents, including *M. bovis* [9,14,15,24]. Hazelton et al. [13], and Gabinaitiene et al. [36] demonstrated that calves fed mastitic milk from cows infected with *M. bovis* are at a higher risk of becoming infected themselves. The association between feeding waste milk and higher *M. bovis* seroprevalence in the current study underscores the role of this practice in the spread of infection from the lactating cohort to the youngstock cohort within herds. It was surprising that feeding waste milk was not detected as associated with seropositivity in spring 1. This may reflect the time lag required between ingestion of a sufficient ‘dose’ of *M. bovis* infected colostrum/milk and expression of a detectable systemic antibody response. Many of the heifers at this visit period may not have been exposed to waste milk feeding practices for sufficiently long enough and may have been too young for detection of an antibody response (median age of the sampled heifers; 1.4 months old). Antibodies against *M. bovis* are not detected until 10–14 days post-infection but detectable levels of antibodies remain for several months to years [25,37,38], hence associations in spring 2 and autumn 2. Culling mastitic cows and either discarding or pasteurising infected colostrum and raw milk are, therefore, recommended to mitigate the risk of transmitting *M. bovis* to calves [39]. Pasteurisation of colostrum or waste milk is not commonly carried out on Irish dairy farms.

Shared airspace between calves and older animals was identified as a potential risk factor for *M. bovis* seropositivity, with a trend toward increased seropositivity in the studied herds. Although not always statistically significant, there was a consistent association across different visit periods and in both models. These findings are consistent with those reported by Gille et al. [10], who demonstrated that co-mingling of animals of different ages or groups, particularly in environments with suboptimal biosecurity, was a risk factor for increasing *M. bovis* transmission.

Older animals, particularly those that are sub-clinically infected or have recovered from clinical *M. bovis* infections, can serve as reservoirs of infection. *M. bovis* is primarily transmitted through respiratory secretions and close contact, making shared airspace, particularly in confined environments such as sheds, a critical risk factor for *M. bovis* transmission [40]. On farms where airspace is shared between animals of different age groups, there may be further shared environmental spaces, such as feeding areas, water troughs, and bedding. In these common areas, the potential for contamination with *M. bovis* through respiratory secretions or nasal discharge is considerable, potentially resulting in a higher pathogen load in the environment and a resultant increased risk of transmitting the infection to calves. In Irish spring-calving dairy systems, cows typically calve indoors during the early spring months. After birth, calves are kept indoors for approximately eight to twelve weeks. Calves commonly share the same airspace as the cows or, in some cases, the previous year’s calves (now yearlings). This shared housing arrangement can pose challenges for disease control, particularly respiratory infections, due to the proximity and potential exposure to pathogens circulating among the older animals. Proper ventilation and separation are critical to minimising these risks, but in many cases, practical farm layouts mean calves are housed in close proximity to older animals, potentially exacerbating disease transmission. To reduce *M. bovis* transmission, calves should be housed separately from older cattle. Adequate ventilation, reduced stocking densities, and physical separation between calf and adult housing, can significantly lower *M. bovis* transmission risk [23,31].

The number of colostrum feeds was found to have a protective effect against *M. bovis* seropositivity, with calves receiving more colostrum feeds showing lower odds of seropositivity across multiple visit periods. This aligns with existing research that highlights the critical role of colostrum in providing passive immunity to newborn calves. Colostrum contains high levels of immunoglobulins, which are essential for the early development of the immune system in calves [41]. Studies have consistently shown that inadequate or delayed colostrum intake significantly increases susceptibility to respiratory infections and systemic diseases such as *M. bovis* [42]. Thus, ensuring that calves receive an adequate volume of high-quality colostrum within the first hours of life is critical for enhancing immunity and reducing the risk of pathogen transmission in herds.

The practice of housing heifer calves separately from bull calves was significantly associated with *M. bovis* seroprevalence in both models. This may indicate a response by such farmers to reduce the risk of transmitting respiratory infections on their farms, some of which may be due to *M bovis* infection. However, as the initial source of *M bovis* infection in young calves is from colostrum/milk and the calving environment, subsequent separation of the heifer and bull calves may be less effective in reducing infection risk.

Other farm management practices.

Navel disinfection was associated with a tendency towards a higher likelihood of *M. bovis* seropositivity during spring 1. *M. bovis* may be transmitted indirectly between a cow and her environment and her calf shortly after birth when the farmer has calved the cow and then handled the calf to disinfect its navel. The open umbilicus is a conduit for pathogens causing omphalitis [43] but possibly also for other maternal or environmental pathogens, including *M. bovis*, introduced as contaminants. The positive association of navel disinfection with *M. bovis* seropositivity may also indicate broader hygiene issues on farms implementing this protocol.

The current study did not identify a significant association between contract heifer rearing and *M. bovis* seropositivity. One possible explanation for the lack of association observed in this study could be the variability in *M. bovis* infection risks across different contract-rearing facilities. Previous studies indicate that while certain facilities have higher infection rates, this risk is not consistent across all operations. Differences in management practices, biosecurity measures, and the presence of asymptomatic carriers could vary significantly between facilities, potentially masking any clear association within the study population [13].

### 4.4. Model Comparison

During visit 1 (spring 1), both Model ≥ 1POS and Model ≥ 3POS show relatively low R-squared values (0.11 and 0.10, respectively), indicating a modest ability to explain the variance in seropositivity. In visit 2 (spring 2), however, Model ≥ 3POS demonstrates a substantially better fit (R-squared = 0.31) compared to Model ≥ 1POS (R-squared = 0.13), suggesting that a stricter criterion of three or more positive heifers improves the model’s ability to capture the dynamics of seropositivity during this period.

Conversely, in visit 3 (autumn 2), both models performed equally well, with identical R-squared values of 0.16, implying that the choice of threshold (one or three positive heifers) has no significant impact on model fit for this period. Overall, these results suggest that while the stricter criterion enhances model performance in certain contexts, particularly in visit 2, its advantage may be less pronounced in other periods.

Hughes et al. [44] demonstrated that McFadden’s R-squared values for logistic regression are generally lower than R-squared values for linear models. Values between 0.2 and 0.4 are considered to indicate a good model fit in epidemiological models of disease risk. The McFadden R-squared values reported for Model ≥ 3POS, ranging from 0.16 to 0.31 across visit periods, suggest a reasonably good fit, particularly in visit 2 (spring 2), where the value reaches 0.31. This indicates that Model ≥ 3POS is better at capturing key infection risk factors, particularly in certain periods, compared to Model ≥ 1POS, which has consistently lower R-squared values (0.11 to 0.16).

The multivariable analysis reveals that both models consistently identified key risk factors, such as the number of land parcels farmed, the purchase of cattle, and the number of colostrum feeds as significant across all visit periods. This consistency highlights the importance of management practices and environmental exposures in influencing *M. bovis* transmission. Model ≥ 3POS tended to capture more severe infection risk factors and exhibited stronger predictive performance overall. Meanwhile, Model ≥ 1POS identified broader risk patterns across herds with varying levels of infection. The results suggest that stricter thresholds for defining herd seropositivity (as in Model ≥ 3POS) may be more effective in detecting higher-risk herds.

### 4.5. Study Limitations

This study has specific limitations that may constrain the broader applicability and interpretation of its results. One key limitation is the potential for selection bias in herd recruitment, as the farms were initially recruited for a separate study assessing the health impacts of contract-rearing of replacement heifers, though a substantial number of these were control herds. As a result, recruited herds may not fully represent the national dairy herd population. Furthermore, the voluntary nature of participation could have resulted in an over-representation of farmers with a greater interest in biosecurity and animal health, potentially skewing the results.

The initial sample size for the contract-rearing study was calculated based on the power requirements specifically needed to assess differences in age at first calving (AFC) between contract-reared and home-farm-reared heifers. Consequently, the sample size may not have been optimally designed to assess the seroprevalence of *M. bovis* at herd-level and it may have limited power for detecting significant associations between risk factors and *M. bovis* seropositivity. This could potentially affect the robustness and external validity of the findings related to *M. bovis* infection, highlighting a need for cautious interpretation and possibly further targeted studies with appropriate power calculations focused on *M. bovis* seroprevalence.

Additionally, a complete dataset was not available for every farm at each visit period (*M. bovis* ELISA results were available for 105 farms at all three farm visit periods; 120 farms were initially enrolled in this study). Incomplete datasets were predominantly a result of missing biosecurity and farm management variables, which limited the ability to comprehensively assess the associations between risk factors and *M. bovis* seropositivity across all farms, potentially weakening the statistical power of this study. Furthermore, the biosecurity and farm management data were collected through self-reported questionnaires, which carry the risk of biases, such as recency and social desirability bias, and may not reflect the actual practices on farms. In addition, biosecurity data were collected at one time point in 2018, which does not account for any subsequent changes in management practices during the study period.

This study followed a single cohort of heifers longitudinally from birth to their first lactation, but contemporaneous, unrecorded, variations in farm management, environmental conditions, and disease challenges could have influenced the results. The blood sampling was limited to 10 randomly selected heifers per herd, which may not fully represent the infection status of the entire herd or heifer cohort. Additionally, this study faced a considerable loss to follow-up, with data available for fewer heifers than initially enrolled, which may have introduced bias if the lost heifers differed in their exposure to risk factors compared to those that remained in this study. These factors collectively necessitate a cautious interpretation of the findings.

Despite the limitations of this study, it significantly advances our understanding of the epidemiology of *M. bovis* in dairy herds. This study identifies several herd-level risk factors, such as the number of land parcels farmed, purchase of cattle, and number of colostrum feeds as predictors of *M. bovis* seropositivity in replacement heifer cohorts. These findings are particularly important for guiding the development of targeted biosecurity measures aimed at controlling *M. bovis* transmission in youngstock. By identifying risk factors associated with *M. bovis* seroprevalence, this study contributes to ongoing efforts to mitigate the impact of this globally significant pathogen in the dairy industry.

## 5. Conclusions

In conclusion, this study provides valuable insights into the factors associated with *M. bovis* seropositivity in replacement dairy heifers at different time points during the rearing period. The findings consistently highlight key risk factors influencing *M. bovis* seropositivity, including the degree of land fragmentation, purchase of cattle, and specific management practices, such as feeding waste milk and shared airspace between calves and older animals. The results underline the importance of herd-level interventions, robust biosecurity protocols, and targeted management strategies in mitigating the spread of *M. bovis* within dairy operations. Future research should aim to address the gaps identified, further refining our understanding of risk factors and optimising control measures for this pathogen.

## Figures and Tables

**Table 1:** Farm management and associated variables included in questionnaires administered to participating dairy farmers.

Questionnaire Section	Variables
Farmer and herd characteristics	Address, herd number, herd designator, reason for participation, calving pattern, herd size, replacement rate, farm enterprise and other stock kept on the farm, herd biosecurity status, number of land blocks managed.
Bioexclusion practices	Use of own equipment/contractor to spread slurry, use of slurry from other herds, grazing management after slurry application. Use of footbaths, livestock transportation method, cleaning/disinfection practices for farm visitors, co-grazing of animals with other species, hygiene practices when working with animals of different ages, rodent control policy, and farm water source. Number of animals purchased and source of bought-in animals, isolation and testing policy for bought-in animals. Details of bounding farms and rivers/streams running through farms. Pets are kept on farms, and wildlife is seen on farms.
Calving and newborn calf management	Type of calving facility used, max number of animals it can house, when cows are moved to calving pen, location of calving facilities, use of calving facilities for sick animals, cleaning and disinfection procedures of calving facilities. Navel disinfection practices, product, and timing of application. Colostrum feeding practices; source, quantity, and method of feeding; assessment of quality and storage.
Unweaned heifer management	Type of milk fed to calves, number of feedings per day, feeding of nonsaleable milk, calf housing, sick calf housing, calf feeding equipment, cleaning/disinfection of housing facilities, weaning criteria.
Weaned heifer management	Grazing management and parasite control strategies.
Herd vaccination protocol (biocontainment practices)	Products used and dates of vaccination for the following: BVD ^1^, IBR ^2^, calf pneumonia, calf diarrhoea, leptospirosis, salmonellosis.
Specific questions for source dairy farmers	Age/weight/month heifers moved to CR responsibility for breeding management, health checks performed on heifers before moving, transport of heifers to and from CR, isolation of heifers upon return from CR.

^1^ BVD: Bovine viral diarrhoea; ^2^ IBR: Infectious bovine rhinotracheitis.

**Table 2:** Median age (in days) of heifer cohort and sampled heifers at each sampling period (n = 105 herds).

Visit Period	Heifers (n)	Heifer Cohort Age (days)	Sampled Heifer Cohort Age (days)
V1 (Spring 2018)	1065	41 (range 1–112)	45 (1–103)
V2 (Spring 2019)	1091	387 (range 265–456)	388 (304–456)
V3 (Autumn 2019)	1090	613 (range 481–694)	613 (483–691)

**Table 3:** Distribution of herds with at least one *M. bovis* seropositive heifer and at least 3 seropositive heifers across three farm visit periods between spring 2018 and autumn 2019 (n = 105 herds).

	Seropositivity Criteria	
	≥1 Positive heifer	≥3 Positive heifers
Visit period	Herd seroprevalence (%) (95% CI)	Herd seroprevalence (%) (95% CI)
V1 (Spring 2018)	50.4 (40.5–60.3)	31.4 (22.7–41.2)
V2 (Spring 2019)	35.2 (26.2–45.1)	32.4 (23.6–42.2)
V3 (Autumn 2019)	45.7 (36.0–55.7)	42.9 (33.2–52.9)

**Table 4:** Mean (se) within-herd *M. bovis* apparent seroprevalence and standard error across three visit periods (10 heifers sampled per herd).

Visit Period	Herds (n = 48 CF, 57 SDF *)	Herds with at Least One *M. bovis*-Positive Heifer (n)	Mean (95% CI) *M. bovis* Seropositivity (%)
V1 (Spring 2018)	105	53	38.3 (31.5–45.1)
V2 (Spring 2019)	105	37	81.3 (72.1–90.5)
V3 (Autumn 2019)	105	48	77.1 (69.0–85.2)

* CF: Control farm: rearing their own heifers, SDF: Source dairy farm: engage in contract-rearing of heifers.

**Table 5:** Animal-level seroprevalence of *M. bovis* in heifers over three farm visit periods. The table shows the number of heifers sampled, the number testing positive for *M. bovis* antibodies, and the corresponding seropositivity percentages for spring 2018 (V1), spring 2019 (V3), and autumn 2019 (V3) (n = 105 herds).

Visit Period	Heifers (n)	Positive *M. bovis* ELISA (n)	*M. bovis* Seropositivity (%) (95% CI)
V1 (Spring 2018)	1065	214	20.1 (17.7–22.5)
V2 (Spring 2019)	1091	319	29.2 (26.5–31.9)
V3 (Autumn 2019)	1090	393	36.1 (33.2–38.9)

**Table 6:** Number of herds classified as *M. bovis* seropositive or seronegative by two seropositivity criteria, across three farm visit periods (n = 105).

Herd *M. bovis* Serostatus Category			Herds (n)	
Visit 1	Visit 2	Visit 3	≥1 Positive Heifer	≥3 Positive Heifers
Positive	Positive	Negative	0	0
Negative	Positive	Negative	0	0
Positive	Negative	Positive	3	1
Negative	Negative	Positive	8	10
Positive	Negative	Negative	23	14
Negative	Positive	Positive	10	16
Negative	Negative	Negative	34	46
Positive	Positive	Positive	27	18

Key: visit 1 (spring 2018), visit 2 (spring 2018), visit 3 (autumn 2019).

**Table 7:** Output from multivariable analysis of variables associated with heifer cohort *M. bovis* seropositivity across three farm visit periods (spring 1, spring 2, and autumn 2) in two models with different criteria for heifer cohort seropositivity. These variables represent the final selection after univariable analysis.

	Model ≥ 1POS: One or More ELISA Positive Heifers Indicate Herd Seropositivity					Model ≥ 3POS: Three or More ELISA Positive Heifers Indicate Herd Seropositivity				
Visit Period	Variable Name	Estimate	SE	Odds ratio (95% CI)	*p* value	Variable Name	Estimate	SE	Odds ratio (95% CI)	*p* value
V1: Spring 1	(n = 99 herds, 46P, 53N, 46.46% seropositivity)					(n = 99 herds, 68N, 31P, 31.3% seropositivity)				
	Number of feeds of colostrum before whole milk or milk	−0.22	0.11	0.81 (0.64, 0.99)	0.05	Other animals kept on the farm (Reference category: No)	1.42	0.62	4.13 (1.24, 14.56)	0.02
	Pre-weaning individual housing only (Reference category: No)	0.85	0.53	2.33 (0.84, 6.93)	0.11	Pre-weaning individual housing only (Reference category: No)	1.09	0.60	2.98 (0.92, 10.06)	0.07
	Heifer calves are housed separately from bull calves (Reference category: No)	0.95	0.47	2.60 (1.05, 6.78)	0.04	Herd size in 2018	0.005	0.00	1.0 (1.0, 1.01)	0.12
	Navel disinfection carried out (Reference category: No)	1.18	0.77	3.25 (0.79, 17.18)	0.12					
	Number of parcels of land farmed during 2018 (Reference category: 3 or less)	0.74	0.49	2.10 (0.82, 5.60)	0.13	Calves share airspace with older animals (Yes/No)	0.76	0.49	2.13 (0.82, 5.76)	0.12
V2: Spring 2	(n = 101 herds, 66N, 35P, 34.65% seropositivity)					(n = 99 herds, 68N, 31P, 31.3% seropositivity)				
	Feed waste milk to calves (Reference category: No)	1.08	0.65	2.93 (0.89, 1.84)	0.10	Feed waste milk to calves (Reference category: No)	1.04	0.68	2.84 (0.81, 12.33)	0.13
	Number of parcels of land farmed during 2018 (Reference category: 3 or less)	0.66	0.51	1.93 (0.71, 5.37)	0.20	Number of parcels of land farmed during 2018 (Reference category: 3 or less)	1.28	0.52	3.59 (1.33, 10.22)	0.01
	Purchased cattle during 2018 (Reference category: No)	1.35	0.60	3.84 (1.27, 13.78)	0.02	Purchased cattle during 2018 (Reference category: No)	1.22	0.62	3.40 (1.09, 12.60)	0.05
	Use group calving pens only (Reference category: No)	0.75	0.48	2.12 (0.84, 5.50)	0.12	Number of feeds of colostrum before whole milk or milk	−0.28	0.13	0.76 (0.57, 0.96)	0.04
	Implemented a vaccination protocol for IBR in calves during 2018 (Reference category: No)	0.77	0.47	2.16 (0.88, 5.48)	0.10					
Autumn 2	(n = 103 herds, 60N, 43P, 46% seropositivity)					(n = 100, 60N, 40P, 40% seropositivity)				
	Calves share airspace with older animals (Reference category: No)	0.84	0.47	2.31 (0.94, 5.97)	0.07	Calves share airspace with older animals (Reference category: No)	0.94	0.49	2.56 (1.0, 1.23)	0.05
	Purchased cattle during 2018 (Reference category: No)	1.22	0.56	3.40 (1.20, 10.86)	0.03	Purchased cattle during 2018 (Reference category: No)	1.31	0.56	3.69 (1.30, 11.92)	0.02
	Heifer calves are housed separately from bull calves (Reference category: No)	1.30	0.50	3.66 (1.43, 10.20)	0.0092	Heifer calves are housed separately from bull calves (Reference category: No)	1.22	0.50	3.40 (1.31, 9.50)	0.01
	Colostrum quality assessed (Reference category: No)	0.90	0.46	2.44 (1.01, 6.13)	0.05	Colostrum from own dam only (Reference category: No)	−0.97	0.63	0.38 (0.10, 1.24)	0.12
	Feed waste milk to calves (Reference category: No)	0.83	0.60	2.30 (0.74, 7.95)	0.16	Number of parcels of land farmed during 2018 (Reference category: 3 or less)	1.14	0.51	3.13 (1.17, 8.91)	0.03
						Have a dedicated sick pen for calves (Reference category: No)	0.73	0.52	2.08 (0.77, 6.02)	0.16

Key: V1: visit 1 (spring 2018), V2: visit 2 (spring 2018), V3: visit 3 (autumn 2019).

**Table 8:** McFadden R-squared values comparing multivariable model performance across visit periods. Model ≥ 1POS indicates herd seropositivity based on one or more positive heifers, while Model ≥ 3POS is based on three or more positive heifers. Higher R-squared values reflect better model fit, showing the ability of the models to explain variance in *M. bovis* seropositivity across different visit periods.

Visit Period	Model ≥ 1POS	Model ≥ 3POS
Visit 1 (Spring 1)	0.11	0.10
Visit 2 (Spring 2)	0.13	0.31
Visit 3 (Autumn 2)	0.16	0.16

## Data Availability

The data used to support the findings of this study are included within the article, and the data are available from the corresponding author upon reasonable request.

## Supplementary Materials

The following supporting information can be downloaded at: https://www.mdpi.com/article/10.3390/ani14213057/s1, Table S1: Output from univariable analysis of variables associated with heifer cohort *M. bovis* seropositivity across three farm visit periods (spring 1, spring 2 and autumn 2) in two models with different criteria for cohort seropositivity (at a significance level of approximately *p* < 0.2). These variables were subsequently used to build the multivariable models.

## Abbreviations

*M. bovis*—*Mycoplasma bovis*; CF—control farm; SDF—source dairy farm; ELISA—enzyme-linked immunosorbent assay; CI—confidence interval; OR—odds ratio; BVD—bovine viral diarrhoea; IBR—infectious bovine rhinotracheitis; BTM—bulk tank milk; ICBF—Irish Cattle Breeding Federation; PCR—polymerase chain reaction; MAP—*Mycobacterium avium* subsp. *paratuberculosis*; SE—standard error; HPRA—Health Products Regulatory Authority.
