# Supplementary material for: Herd-Level Risk Factors Associated with Mycoplasma bovis Serostatus in Youngstock on Irish Dairy Farms
Source: Animals (Basel). 2024 Oct 23;14(21):3057. doi: 10.3390/ani14213057 (PMC11545067; doi:10.3390/ani14213057)
Supplement: Supplementary file 1 [file animals-14-03057-s001.zip › animals-3244038-supplementary materials.pdf]

Supplementary material

Table S1. Output from univariable analysis of variables associated with heifer cohort *M. bovis* seropositivity across three farm visit periods (spring 1, spring 2 and autumn 2) in two models with different criteria for cohort seropositivity (at a significance level of approximately $p < 0.2$). These variables were subsequently used to build the multivariable models.

Visit period	Model ≥ 1POS (Herd seropositive if ≥ 1 ELISA positive heifer)	Estimate	P value	Model ≥ 3POS (Herd seropositive if ≥ 3 ELISA positive heifers)	Estimate	P value
V1: Spring 1	Heifers housed separate from bulls (Referent category: No)	0.78	0.06	Number of feeds colostrum before whole milk or milk replacer	-0.17	0.13
	Number of feeds colostrum before whole milk or milk replacer	-0.19	0.05	Other animals kept on the farm (Referent category: No)	0.89	0.07
	Navel disinfection carried out (Referent category: No)	0.97	0.12	Number of blocks of land farmed during 2018 (Referent category: 3 or less)	0.58	0.19
	Herd size during 2018 (Number of females over 2 years September 2018)	0.003	0.05	Pre-weaning individual housing only (Referent category: No)	0.60	0.21
	Calves exposed to cow manure (Referent category: No)	-1.16	0.09			
	Colostrum from own dam only (Referent category: No)	-0.59	0.23			
V2: Spring 2	Colostrum quality assessed (Referent category: No)	0.65	0.12	Number of feeds colostrum before whole milk or milk replacer	-0.18	0.11

	Feed waste milk to calves (Referent category: No)	1.07	0.07	Feed waste milk to calves (Referent category: No)	0.92	0.12
	Purchased cattle during 2018 (Referent category: No)	1.0	0.05	Purchase cattle during 2018 (Referent category: No)	0.83	0.11
	Number of blocks of land farmed during 2018 (Referent category: 3 or less)	0.72	0.1	Number of blocks of land farmed during 2018 (Referent category: 3 or less)	0.92	0.04
	Dedicated sick pen for calves (Referent category: No)	0.61	0.18	Calves share airspace with older animals (Referent category: No)	0.55	0.2
	Use group calving pens only (Referent category: No)	0.76	0.07	Use group calving pens only (Referent category: No)	0.70	0.1
V3: Autumn 2	Median percentage of animals with abnormal nasal scores at spring 1 visit	6.38	0.19	Heifer calves are housed separately from bull calves (Referent category: No)	0.82	0.06
	10 calves or less in group calf pens (Referent category: No)	-0.54	0.19	Number of feeds colostrum before whole milk or milk replacer	-0.13	0.18

Heifer calves are housed separate from bull calves Referent category: No)	0.65	0.12	Feed waste milk to calves (Referent category: No)	0.70	0.18
Colostrum quality assessed (Referent category: No)	0.79	0.05	Purchased cattle during 2018 (Referent category: No)	0.79	0.09
Feed waste milk to calves (Referent category: No)	0.85	0.1	Number of blocks of land farmed during 2018 (Referent category: 3 or less)	0.73	0.09
Breeding protocol for cows (Referent category: AI only)	0.77	0.14	Colostrum from own dam only (Referent category: No)	-0.70	0.18
Farm category (Referent category: Contract-rearing)	0.54	0.16	Calves share airspace with older animals (Referent category: No)	0.52	0.21
Purchased cattle during 2018 (Referent category: No)	0.95	0.04	Dedicated sick pen used for calves (Referent category: No)	0.70	0.11
Number of blocks of land farmed during 2018 (Referent category: 3 or less)	0.57	0.19	Use group calving pens only (Referent category: No)	0.65	0.11

Change gloves between different age groups (Referent category: No)	0.55	0.17	
Calves exposed to cow manure (Referent category: No)	-0.94	0.17	
Calves share airspace with older animals (Referent category: No)	0.66	0.11	
Dedicated sick pen used for calves (Referent category: No)	0.68	0.12	
Use group calving pens only (Referent category: No)	0.56	0.16	
